# H3K4 trimethylation by CclA regulates pathogenicity and the production of three families of terpenoid secondary metabolites in *Colletotrichum higginsianum*

**DOI:** 10.1111/mpp.12795

**Published:** 2019-03-29

**Authors:** Jean‐Félix Dallery, Émilie Adelin, Géraldine Le Goff, Sandrine Pigné, Annie Auger, Jamal Ouazzani, Richard J. O'Connell

**Affiliations:** ^1^ UMR BIOGER, INRA, AgroParisTech Université Paris‐Saclay 78850 Thiverval‐Grignon France; ^2^ Centre National de la Recherche Scientifique Institut de Chimie des Substances Naturelles ICSN Avenue de la Terrasse 91198 Gif‐sur‐Yvette, cedex France

**Keywords:** colletochlorin, *Colletotrichum*, COMPASS, higginsianin, histone methylation, pathogenicity, sclerosporide

## Abstract

The role of histone 3 lysine 4 (H3K4) methylation is poorly understood in plant pathogenic fungi. Here, we analysed the function of CclA, a subunit of the COMPASS complex mediating H3K4 methylation, in the brassica anthracnose pathogen *Colletotrichum higginsianum*. We show that CclA is required for full genome‐wide H3K4 trimethylation. The deletion of *cclA* strongly reduced mycelial growth, asexual sporulation and spore germination but did not impair the morphogenesis of specialized infection structures (appressoria and biotrophic hyphae). Virulence of the Δ*cclA* mutant on plants was strongly attenuated, associated with a marked reduction in appressorial penetration ability on both plants and inert cellophane membranes. The secondary metabolite profile of the Δ*cclA* mutant was greatly enriched compared to that of the wild type, with three different families of terpenoid compounds being overproduced by the mutant, namely the colletochlorins, higginsianins and sclerosporide. These included five novel molecules that were produced exclusively by the Δ*cclA* mutant: colletorin D, colletorin D acid, higginsianin C, 13‐*epi*‐higginsianin C and sclerosporide. Taken together, our findings indicate that H3K4 trimethylation plays a critical role in regulating fungal growth, development, pathogenicity and secondary metabolism in *C. higginsianum*.

## Introduction

Fungi are rich sources of bioactive natural products (mostly secondary metabolites) with high value for the pharmaceutical industry (e.g. paclitaxel, penicillin, cyclosporins and statins) (Andersen *et al*., 2013; Wiemann and Keller, 2014). In addition, many secondary metabolites produced by plant pathogenic fungi have important economic impacts through their toxicity to humans and animals (mycotoxins in foodstuffs) and plants (phytotoxins) (Wu *et al*., 2014; Mobius and Hertweck, 2009). In fungal genomes, the genes required for synthesis of a particular metabolite are typically grouped in biosynthetic gene clusters (BGCs), which can be computationally predicted (Weber *et al*., 2015). Based on the wealth of genomic data now available for a broad range of fungal taxa, it is clear that the capacity of filamentous fungi to elaborate secondary metabolites has been significantly underestimated. Thus, in most species many more BGCs can be identified than the small number of secondary metabolites that are produced under standard laboratory conditions (Andersen *et al*., 2013). Over the last decade, intensive efforts have been directed towards unlocking the cryptic secondary metabolome of fungi using a variety of approaches, including heterologous expression (i.e. constitutive or inducible expression of genes in a host fungus), the use of culture media that mimic the natural environment and genetic or chemical manipulation of chromatin remodelling factors (Gacek and Strauss, 2012; Wiemann and Keller, 2014).

Alteration of the chromatin landscape by genetic manipulation of histone‐modifying enzymes has proven to be a productive approach to identify new secondary metabolism (SM) products in numerous fungi (Chiang *et al*., 2009; Brakhage, 2013; Wu and Yu, 2015). Most histone‐modifying enzymes investigated to date are histone methyltransferases (HMTs) (e.g. Studt *et al*., 2016a), histone acetyltransferases (HATs) (e.g. Fan *et al*., 2017), histone deacetylases (HDACs) (e.g. Wu *et al*., 2016) and histone demethylases (HDMs) (e.g. Gacek‐Matthews *et al*., 2015). For example, in *Epichloe festucae*, the loss of trimethylation of histone protein H3 at lysine 9 (H3K9me3) or lysine 27 (H3K27me3) in the promoter regions of SM genes activated their expression *in vitro* and was also needed for the mutualistic interaction of this endophyte with *Lolium perenne *(Chujo and Scott, 2014). In *Fusarium graminearum*, loss of the H3K27me3 histone mark lead to the activation of 71% of the 45 BGCs during axenic growth (Connolly *et al*., 2013), while in *Fusarium fujikuroi* reduced levels of H3K27 trimethylation were associated with the induction of four silent BGCs and the accumulation of novel metabolites (Studt *et al*., 2016b).

The COMPASS (COMplex of Proteins ASsociated with Set1) complex is conserved from yeast to multicellular eukaryotes and consists of eight proteins (Set1, Bre2, Sdc1, Shg1, Spp1, Swd1, Swd2 and Swd3 in *Saccharomyces cerevisiae*) that collectively mediate the mono‐, di‐ and trimethylation of lysine 4 in histone H3 (Miller *et al*., 2001). Although usually a characteristic of transcriptionally active regions, H3K4 methylation by COMPASS has also been implicated in the transcriptional silencing of genes located near chromosome telomeres in yeast and filamentous fungi (Krogan *et al*., 2002). In three Aspergilli (*A. nidulans*, *A. fumigatus* and *A. oryzae*), the deletion of a COMPASS subunit called CclA (a homologue of *S. cerevisiae* Bre2) resulted in the overproduction of numerous different secondary metabolites (Bok *et al*., 2009; Palmer *et al*., 2013; Shinohara *et al*., 2016). Deletion of a CclA homologue in *Pestalotiopsis fici* led to the discovery of pestaloficiols T to V and ficipyrone C (Wu *et al*., 2016). In contrast, the deletion of FgSet1 and FgBre2 in *F. graminearum* resulted in lower levels of deoxynivalenol *in vitro* and no increase in the production of any other secondary metabolites was reported (Liu *et al*., 2015).

The ascomycete genus *Colletotrichum* contains over 190 recognized species, including many that provoke devastating diseases on monocot and dicot crops worldwide, as well as some endophytic species (Crouch *et al*., 2014, Jayawardena *et al*., 2016). *Colletotrichum higginsianum*, causal agent of crucifer anthracnose disease, is responsible for important economic losses on cultivated members of the Brassicaceae in tropical and subtropical regions but also infects the model plant *Arabidopsis thaliana* (O'Connell *et al*., 2004; Birker *et al*., 2009). The recent re‐sequencing of this fungal genome using single‐molecule real‐time sequencing produced a gapless assembly of the 12 chromosomes (Dallery *et al*., 2017), which facilitated a more accurate annotation of SM genes. The *C. higginsianum* SM gene repertoire was revealed to be one of the largest described to date (Giles *et al*., 2011), comprising 77 predicted BGCs (Dallery *et al*., 2017). Transcriptome sequencing showed that of the 23 BGCs expressed under any of the four tested conditions, the majority (21) were only induced during plant infection and their patterns of expression were highly stage‐specific (Dallery *et al*., 2017). Currently no information is available about the role of histone‐modifying proteins in the control of secondary metabolism in *C. higginsianum* or any other *Colletotrichum* species.

To gain insight into the mechanism of SM regulation in *C. higginsianum*, and in an effort to activate cryptic SM biosynthetic pathways in mycelia grown axenically, we deleted the orthologue of *cclA* in this fungus. The resulting mutants were strongly impaired in their saprophytic growth, conidiation, germination, appressorium penetration and pathogenicity on *Arabidopsis* plants. Comparative metabolite profiling of the ∆*cclA* mutant and wild‐type strain revealed that 11 molecules, including three different families of terpenoid metabolites, were overproduced by the mutant growing axenically. Five novel molecules were only detectable in cultures of the ∆*cclA* mutant, and we describe the full structural characterization of these elsewhere (Dallery *et al*., 2019). Our findings demonstrate that chromatin remodelling mediated by CclA contributes to regulating secondary metabolism and pathogenicity in *C. higginsianum*.

## Results

### Production of a *cclA* targeted deletion mutant and genetic complementation

A homologue of CclA (Bre2 in *Saccharomyces cerevisiae*) was identified in *C. higginsianum* using the *A. nidulans* CclA protein sequence (AN9399) (Bok *et al*., 2009) to query the National Center for Biotechnology Information (NCBI) nr database with blastp. The only *C. higginsianum* hit obtained (CH63R_04661) was then aligned with characterized Bre2 homologues and phylogeny was inferred (Supplementary Fig. S1). This *C. higginsianum* Bre2 homologue is hereafter referred to as CclA.

To produce a targeted deletion mutant, the *cclA* open reading frame (ORF) was replaced by the hygromycin resistance gene using the primers shown in Supplementary Table S1. Correct at‐the‐locus integration was confirmed by diagnostic Polymerase Chain Reaction (PCR) and Southern blots for three independent transformants (Supplementary Fig. S2). One of these transformants (#18) was selected for regeneration of the wild‐type genetic locus, whereby the hygromycin resistance cassette was replaced with the full *cclA* ORF including native promoter and terminator sequences, at the native genomic location. Two independent transformants were selected based on their sensitivity to hygromycin and hereafter these will be referred to as complemented strains C9 and C10.

### CclA is required for full H3K4 trimethylation

**Figure 1:**
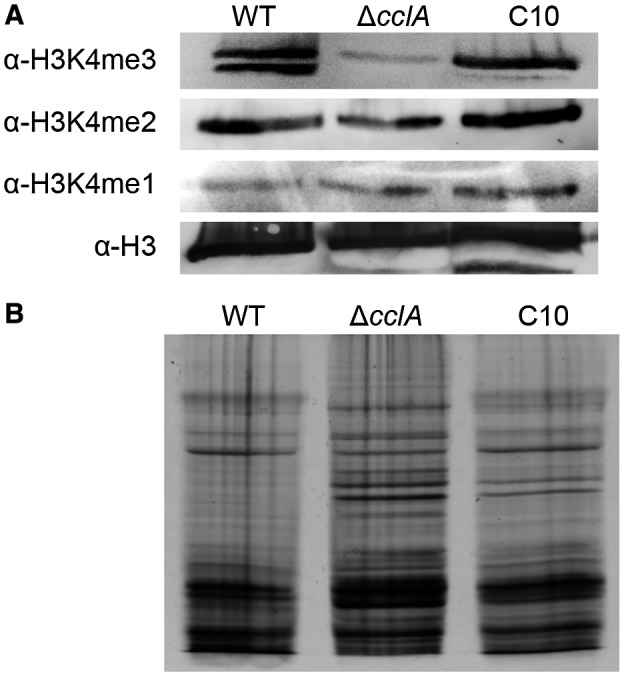
Loss of CclA impairs global H3K4 trimethylation in *Colletotrichum higginsianum*. Global H3K4 methylation status was analysed by western blot of nuclear protein extracts. (A) Histone marks detected with antibodies specific for mono‐, di‐ or trimethylated H3K4 residues. An antibody detecting global H3 proteins was used as a loading control. (B) Coomassie blue staining used as a second loading control.

We investigated the link between CclA and H3K4 methylation status by Western blot analyses of nuclear protein extracts of the wild‐type and mutant strains. The methylation status of the H3K4 residue (mono‐, di‐ or trimethylated) was assayed using antibodies specific for each histone mark (Fig. 1A). Loading controls were performed by immuno‐detection of the histone H3 C‐terminus and with Coomassie blue staining of a second gel run in parallel (Fig. 1B). The methylation level remained unchanged in the ∆*cclA* mutant for both H3K4me and H3K4me2 marks, whereas the level of H3K4 trimethylation was greatly reduced, though not eliminated. H3K4 trimethylation was restored to wild‐type levels in the complemented strain C10 (Fig. 1A). These results show that CclA is required for full H3K4 trimethylation in *C. higginsianum*, as was found previously with CclA/Bre2 mutants in *A. thaliana* (Jiang *et al*., 2011), *Schizosaccharomyces pombe* (Mikheyeva *et al*., 2014), *Aspergillus oryzae* (Shinohara *et al*., 2016) and *Fusarium *spp. (Studt *et al*., 2017).

### Loss of CclA impairs mycelial growth, sporulation and spore germination

**Figure 2:**
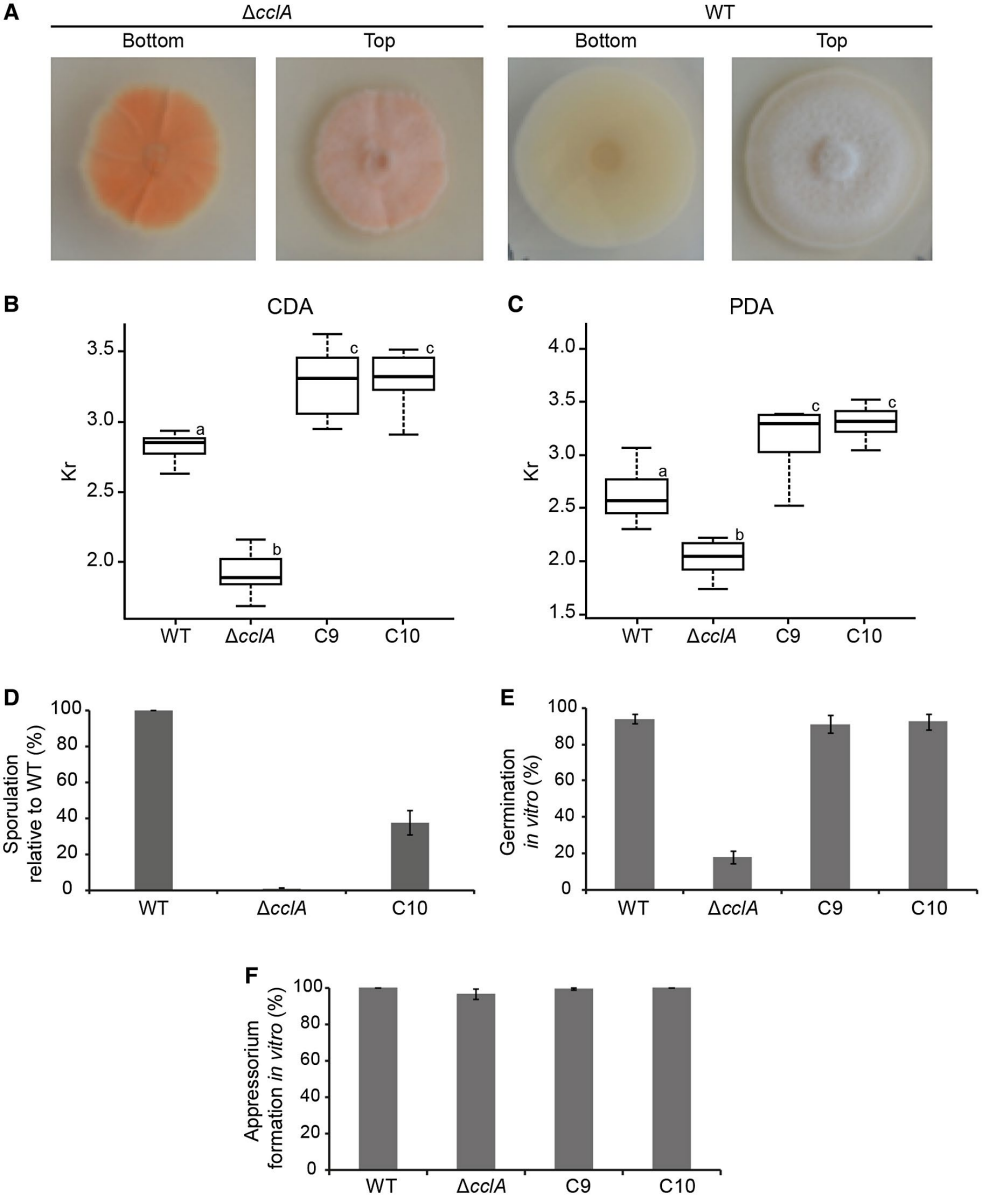
Loss of CclA reduces radial growth, sporulation and germination. (A) Appearance of wild type (WT) and Δ*cclA* mycelia growing on potato dextrose agar (PDA) medium, viewed from below (bottom) or above (top). (B,C) Boxplots showing the growth coefficients (Kr) of WT, Δ*cclA* and complemented strains C9 and C10 on Czapek‐Dox agar (CDA) (B) and PDA (C). Different letters indicate significantly different statistical groups (analysis of variance [ANOVA], *P* < 0.01). (D) Histogram showing the level of sporulation on Mathur's medium of the Δ*cclA* and C10 strains relative to WT. (E) Histogram showing the level of spore germination of WT, Δ*cclA* and complemented strains on polystyrene. (F). Histogram showing the proportion of germinated spores of the WT, Δ*cclA* and complemented strains forming appressoria on polystyrene. (D,E,F) Error bars represent standard deviation (SD).

On potato dextrose agar (PDA), colonies of the ∆*cclA* mutant appeared smaller than those of the wild type and had a strong orange pigmentation (Fig. 2A). To quantify mycelial growth of the fungus in axenic conditions, colony radial growth rate was measured on a complex medium (PDA) and a minimal medium (Czapek‐Dox agar, CDA). Hyphal growth rate was significantly reduced (analysis of variance [ANOVA], *P*‐value < 0.01) in the ∆*cclA* mutant compared to the wild‐type strain on both media (77% and 68% of wild type on PDA and CDA, respectively, Fig. 2B,C). Complementation with a wild‐type copy of the *cclA* gene fully rescued the growth defect and the mycelial growth rates of complemented strains C9 and C10 were not significantly different from each other or the wild‐type strain (Fig. 2B,C). To further investigate the deleterious effect of ∆*cclA *mutation on fungal development, the conidiation rate (Fig. 2D) was assessed on a medium that favours sporulation in most *Colletotrichum* species (Mathur *et al*., 1950). Deletion of *cclA* strongly reduced conidiation to only 0.5% of the wild‐type level. In addition to this sporulation defect, the ∆*cclA* mutant also displayed a larger range of spore shapes and lengths (range 10.7 μm to 20.3 μm) than wild‐type conidia (range 14.5 μm to 17.6 μm) (Supplementary Fig. S3). We then investigated the ability of spores to germinate on artificial hydrophobic (polystyrene) surfaces. The ∆*cclA *mutant was strongly impaired in germination, with only 20% of conidia forming germ‐tubes or appressoria compared to 95% germination by wild‐type conidia, while germination was restored to wild‐type levels in complemented strains C9 and C10 (Fig. 2E). Remarkably, although the ∆*cclA* mutant produced dramatically fewer spores, which germinated poorly, once a spore had formed a germ‐tube, an appressorium subsequently developed at its apex in nearly all cases and with similar frequency to the wild type (Fig. 2F). Moreover, appressoria formed by the Δ*cclA* mutant had the same morphology and level of cell wall melanization as the wild‐type strain, and they developed a basal penetration pore as normal (Supplementary Fig. S4). Thus, appressorium morphogenesis was not visibly impacted by the loss of CclA. However, germinated conidia of the Δ*cclA* mutant frequently produced two appressoria from one spore, or one appressorium plus one germ‐tube (Supplementary Fig. S4B), which is rare in the wild‐type strain (Supplementary Fig. S4A). The subset of aberrantly small conidia (< 14 μm in length) of the Δ*cclA* mutant rarely germinated but those that did germinate formed small, weakly‐melanized appressoria (Supplementary Fig. S4C).

### CclA deletion impairs fungal virulence but not infection‐related morphogenesis

**Figure 3:**
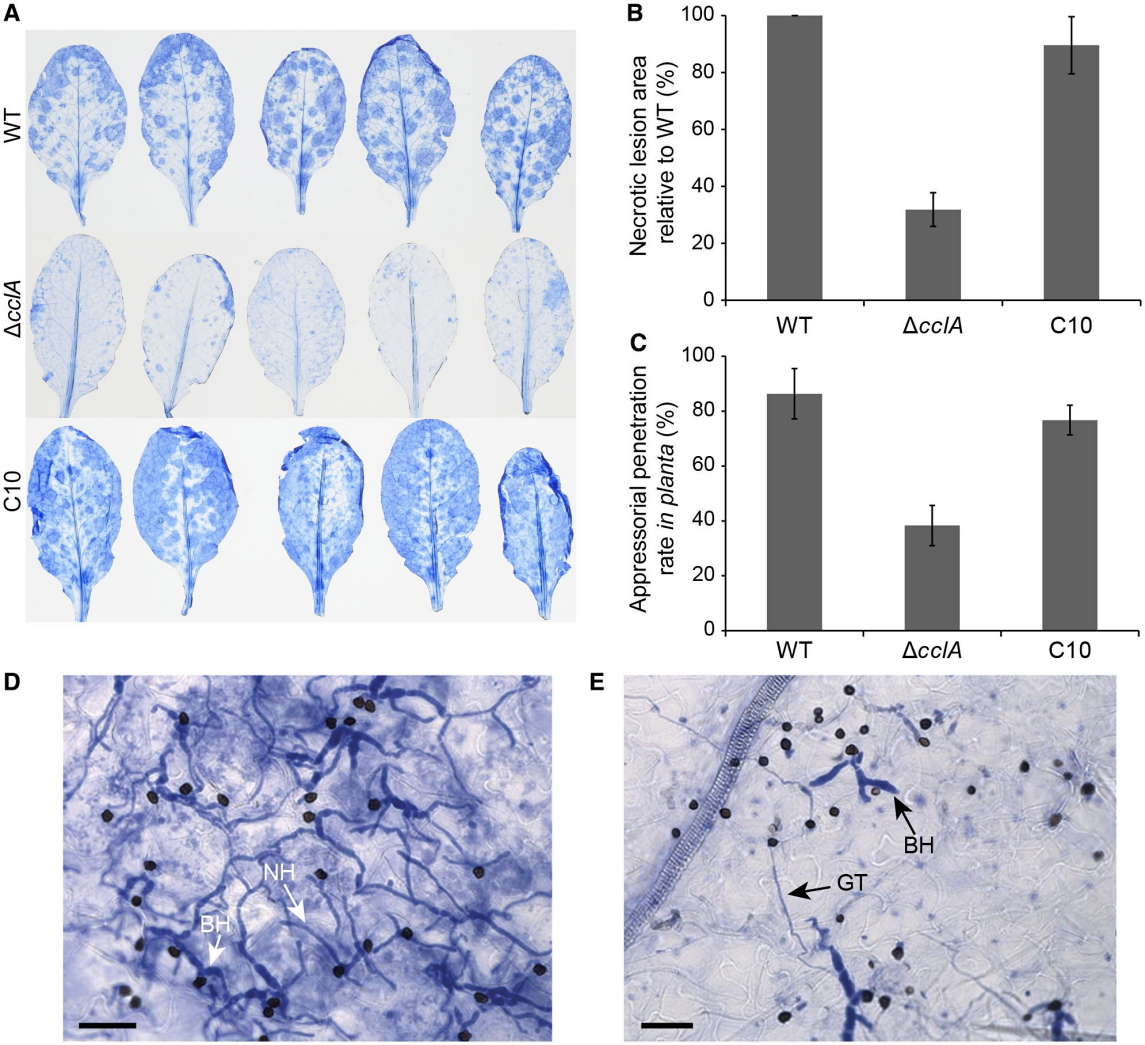
Deletion of *cclA* impairs fungal penetration and virulence on *Arabidopsis* plants. (A) Representative infected leaves were collected at 3 days post‐inoculation (dpi) with the wild type (WT), Δ*cclA* mutant or C10 complemented strain and stained with Trypan blue‐lactophenol to detect fungal mycelium and dead plant cells. (B) Histogram showing necrotic lesion areas stained with Trypan blue quantified using ImageJ. Lesion areas for Δ*cclA* and C10 are shown as a percentage of the WT. Data were combined from three independent inoculation experiments. (C) Histogram showing the appressorial penetration rate quantified by microscopic inspection of Trypan blue‐stained leaves. At least 100 appressoria per leaf were counted, three different leaves per strain. Data were combined from three independent inoculation experiments. (D,E) Bright‐field microscopy images showing *Arabidopsis* leaf tissue infected by the wild type (D) or ∆*cclA* mutant (E) at 3 dpi, and stained with Trypan blue‐lactophenol to reveal fungal hyphae and dead plant cells. (D) Wild‐type appressoria have penetrated with high frequency and produced abundant bulbous biotrophic hyphae (BH) and thinner filamentous necrotrophic hyphae (NH). Many dead infected cells are stained blue. (E) Most ∆*cclA* appressoria have failed to penetrate epidermal cells, and the few penetrated appressoria have produced only BH. Infected host cells are not stained by Trypan blue. Bars = 30 μm. GT = conidial germ‐tube on the leaf surface. (B,C) Error bars represent standard deviation (SD).

To determine the impact of ∆*cclA* deletion on fungal pathogenesis, we quantified the extent of fungal colonization on mature *Arabidopsis* plants using Trypan blue‐lactophenol to stain necrotic lesions (i.e. dead host cells and fungal mycelium) at 3 dpi (Fig. 3A,B). The area of the leaf occupied by necrotic lesions was then quantified by image analysis. The ∆*cclA* mutant presented a marked reduction in pathogenicity, causing only 30% of the wild‐type level of necrosis, whereas this virulence defect was rescued in the complemented strain C10 (Fig. 3A,B). To quantify the appressorial penetration rate, the Trypan blue‐stained leaf tissues were examined by microscopy at 3 dpi. Appressoria were considered to have penetrated when a biotrophic hypha was visible in the underlying epidermal cell. On average, only 39% of ∆*cclA *appressoria successfully penetrated, compared to 86% for the wild‐type strain. Once again, penetration ability was restored in the complemented strain C10 (Fig. 3C). Microscopy of the infected tissues also revealed that most appressoria of the wild type had penetrated to form both bulbous biotrophic hyphae and thin, filamentous necrotrophic hyphae that had extensively colonized the leaf at 3 dpi (Fig. 3D). In striking contrast, the small number of ∆*cclA *appressoria that successfully penetrated had only produced biotrophic hyphae at 3 dpi, although these were similar to wild type in their morphology (Fig. 3E). Moreover, most host cells infected by the wild‐type strain were dead (stained by Trypan blue) whereas those infected by ∆*cclA* were not stained and may therefore have been alive (biotrophic infections).

**Figure 4:**
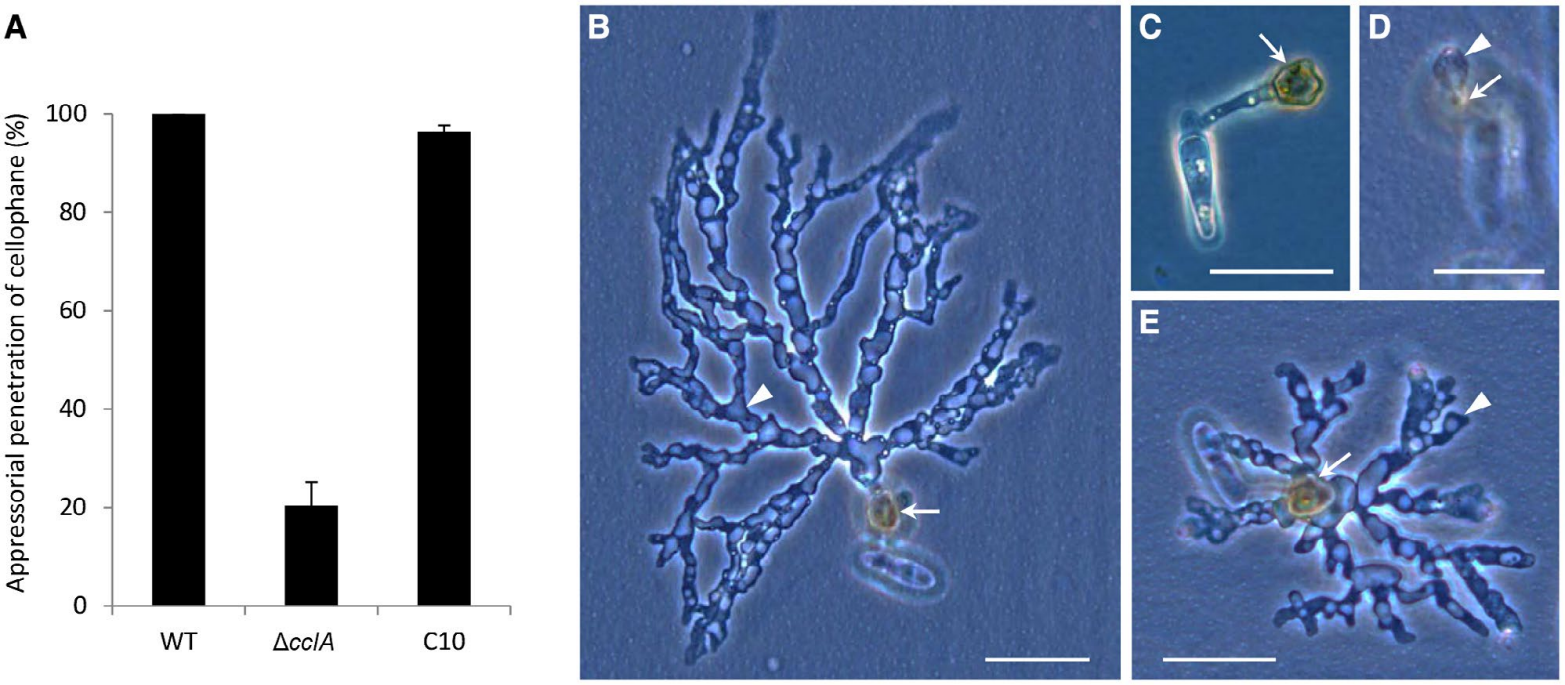
Appressoria of the Δ*cclA* mutant fail to penetrate inert cellophane membranes. (A) Histogram showing the appressorial penetration frequency of the wild‐type strain (WT), *ΔcclA* mutant and C10 complemented strain on cellophane membranes after 48 h. Data shown are mean percentages based on counts of > 100 appressoria per replicate, three replicates per genotype. Error bars = 1 standard error (SE). (B–E) Phase‐contrast micrographs showing the morphology of appressoria (arrows) of the wild‐type strain (B), Δ*cclA* mutant (C,D) and C10 complemented strain (E) penetrating cellophane membranes to form pseudo‐hyphae (arrowheads) inside the membrane. Bars = 20 μm.

In view of the reduced frequency of host penetration shown by appressoria of the Δ*cclA* mutant, we also evaluated the ability of mutant appressoria to penetrate inert cellophane membranes (Fig. 4A). Wild‐type appressoria penetrated cellophane very efficiently (100% penetration after 48 h) and produced extensive, branched networks of pseudo‐hyphae growing inside the membrane (Fig. 4B). In marked contrast, most appressoria of the Δ*cclA* mutant failed to penetrate (Fig. 4C), and the few that did penetrate (20.4%) produced only small hyphae less than the diameter of an appressorium (Fig. 4D). Penetration ability and hyphal invasive growth were both fully restored in the C10 complemented strain (Fig. 4A,E). Thus, the loss of CclA impairs the penetration ability of appressoria in a plant‐independent manner.

### Loss of CclA deregulates production of three families of terpenoid metabolites *in vitro*

**Figure 5:**
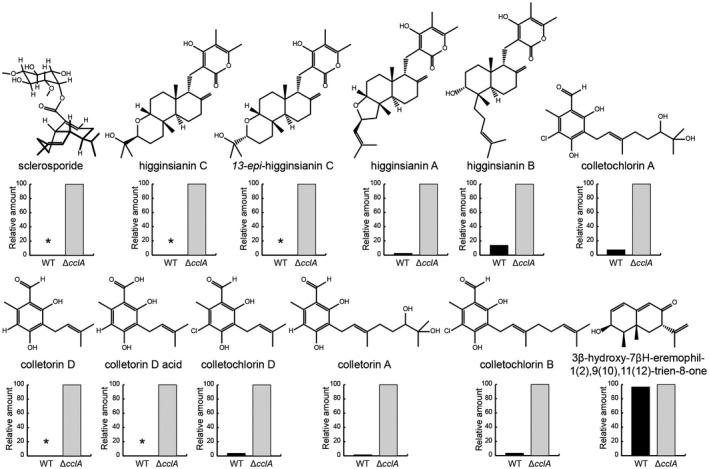
The Δ*cclA* mutant overproduces three families of terpenoid metabolites during axenic growth*.* Structures of molecules isolated from cultures of the wild‐type strain (WT) and Δ*cclA* mutant after 5 days' growth in potato dextrose broth (PDB). The molecules identified belong to four different families, namely the colletochlorins and higginsianins, in addition to the single molecules 3β‐hydroxy‐7βH‐eremophil‐1(2),9(10),11(12)‐trien‐8‐one and sclerosporide. The histograms show the relative abundance of each molecule in the wild type compared to the mutant (taken as 100%). Data were based on measuring peak areas from the high‐performance liquid chromatography (HPLC) chromatograms. Stars indicate molecules that were undetectable in the WT extract.

For comparative metabolite profiling, metabolites produced by the wild‐type and ∆*cclA* strains were adsorbed onto hydrophobic resin beads added to the potato dextrose broth (PDB) during growth and then extracted using organic solvents from the mycelium plus beads harvested from 5‐day‐old cultures. After analysis of the extracts by high‐performance liquid chromatography‐mass spectrometry (HPLC‐MS), peaks were identified by comparison with characterized compounds (Dallery *et al*., 2019), and the relative amount of each molecule was inferred from the HPLC chromatograms. Many compounds that were either not detectable or produced in only small amounts by wild‐type cultures were overproduced by cultures of the ∆*cclA* mutant (Fig. 5, Supplementary Table S2). On the other hand, some molecules present in the wild‐type metabolite profile, such as the sesquiterpene 3β‐hydroxy‐7βH‐eremophil‐1(2),9(10),11(12)‐trien‐8‐one, were not overproduced in the ∆*cclA *mutant (Fig. 5, Supplementary Table S2). In‐depth chemical analyses including nuclear magnetic resonance (NMR) and crystallography revealed that three distinct families of terpenoids were overproduced in the ∆*cclA* mutant, namely the colletochlorins, higginsianins and sclerosporide. The colletochlorin family comprised the previously described colletorin A and colletochlorins A, B and D (Kosuge *et al*., 1973, Kosuge *et al*., 1974a, 1974b) and two novel compounds, colletorin D and colletorin D acid (Fig. 5). The higginsianin family comprised the previously reported higginsianins A and B (Cimmino *et al*., 2016) and two new analogues, higginsianin C and 13‐*epi*‐higginsianin C. Sclerosporide, a novel inositol‐conjugated form of sclerosporin, was also detected in the ∆*cclA* mutant. Full structural characterization of the five new molecules is described in detail elsewhere (Dallery *et al*., 2019).

## Discussion

The deletion of *cclA* in *C. higginsianum* strongly reduced hyphal growth, asexual sporulation and spore germination, suggesting that genes involved in these developmental processes require H3K4 trimethylation. Although *A. fumigatus* and *A. nidulans* mutants lacking *cclA* likewise showed a crippled growth phenotype (Palmer *et al*., 2013; Giles *et al*., 2011), this mutation had little effect on hyphal growth or conidiation in *F. graminearum* or *F. fujikuroi* (Studt *et al*., 2017). The severe developmental phenotypes shown by *C. higginsianum* ∆*cclA *more closely resemble those of fungal mutants lacking the COMPASS catalytic subunit, SET1, which performs H3K4 methylation. Thus, *F. graminearum*, *F. fujikuroi* and *Magnaporthe oryzae* *SET1* mutants all showed strongly reduced growth, while the latter two species were in addition defective in conidiation (Liu *et al*., 2015; Pham *et al*., 2015; Janevska *et al*., 2018).

Although the loss of CclA in *C. higginsianum* had a mild effect on conidial morphogenesis it did not visibly impact morphogenesis of the specialized fungal cell types required for plant invasion, namely melanized appressoria and biotrophic hyphae. Nevertheless, the virulence of *C. higginsianum *on *Arabidopsis* plants was markedly reduced in the absence of CclA, as shown by the production of fewer and smaller necrotic lesions and reduced tissue colonization. It is likely that this attenuation of fungal virulence is largely explained by the strong growth defect of the mutant. However, it was striking that not only host epidermal cells but also inert cellophane membranes were penetrated with much lower frequency by appressoria of the ∆*cclA* mutant than those of the wild type. This suggests that H3K4 trimethylation could play a role in regulating genes required for appressorium function or the invasive growth ability of penetration hyphae, rather than the suppression of plant immunity.

To our knowledge, virulence defects were not previously reported for other fungal ∆*cclA* mutants. For example, the deletion of *cclA* did not affect the virulence of either *F. graminearum* or *F. fujikuroi *on wheat and rice, respectively (Studt *et al*., 2017). Nevertheless, in *F. graminearum*, the deletion of *cclA* (*FgBre2*) did reduce production of the virulence factor deoxynivalenol (DON) *in vitro* (Liu *et al*., 2015). Likewise in a more recent study, deletion of the same gene, named *FgCcl1*, reduced DON production in culture, and revealed a similar effect on gibberellic acid production in the corresponding *F. fujikuroi* mutant (Studt *et al*., 2017). Remarkably, the production of both virulence factors was rescued when the two mutants infected their respective host plants, suggesting that in *Fusarium* species plant‐derived signals can in some way compensate for the absence of CclA (Studt *et al*., 2017).

Studies on the numerous toxins and other secondary metabolites produced by *Colletotrichum *species were reviewed by (García‐Pajón and Collado, 2003). However, natural products produced by *C. higginsianum* were only reported recently (Cimmino *et al*., 2016; Masi *et al*., 2017a; 2017b). These include the diterpenes subglutinol A and BR‐050, that were previously isolated from *Fusarium subglutinans* (Lee *et al*., 1995) and *Penicillifer superimpositus* (Terumi *et al*., 1995). In 2016, Cimmino and co‐workers reported two epimers of subglutinol A and BR‐050, which they called higginsianin A and B, respectively. Colletochlorin A (Kosuge *et al*., 1973), colletochlorins E‐H, 4‐chloroorcinol (Monde *et al*., 1998), colletopyrone (Gohbara *et al*., 1976) and colletopyrandione were also isolated from the same strain of *C. higginsianum* as that studied here (Masi *et al*., 2017a; 2017b). In the present study, we discovered five novel terpenoid compounds from cultures of the ∆*cclA* mutant, including two additional members of the colletochlorin family (colletorin D and colletorin D acid), two new higginsianin analogues (higginsianin C and 13‐*epi*‐higginsianin C) as well as an inositol‐conjugated form of sclerosporin that we called sclerosporide. Based on the structures of known compounds produced by *Colletotrichum* and these new molecules identified from the ∆*cclA* mutant, we could deduce putative biosynthetic pathways for the colletotochlorin and higginsianin compound families, which are presented elsewhere (Dallery *et al*., 2019). Work is now underway to identify the BGCs responsible for producing these two compound families and to elucidate their role in fungal pathogenicity using reverse genetics.

The di‐ and trimethylation of histone H3 lysine 4 mediated by COMPASS is typically associated with an open euchromatin structure and active gene transcription in eukaryotes (Strauss and Reyes‐Dominguez, 2011). It is therefore unexpected that a reduction in H3K4 methylation caused by loss of the COMPASS component CclA results in increased secondary metabolite production. Nevertheless, the deletion of *cclA* was also found to increase metabolite production in several other filamentous fungi, namely *Pestalotiopsis fici* and three different species of *Aspergillus* (Bok *et al*., 2009; Palmer *et al*., 2013). The mechanism is currently unclear, but it is known that H3K4me2/3 can cause gene repression in budding yeast, where COMPASS is required for silencing the mating type loci, ribosomal DNA and genes located near chromosome telomeres (Mueller *et al*., 2006). In *A. nidulans*, where the deletion of *cclA* activated the expression of at least two silent BGCs, chromatin immunoprecipitation (ChIP) of the cluster genes revealed not only strongly reduced levels of H3K4me2/3 but also lower levels of H3K9me2/3, a heterochromatin mark associated with gene repression (Bok *et al*., 2009). This finding raises the possibility that there may be crosstalk between H3K4 and H3K9 methylation (Strauss and Reyes‐Dominguez, 2011). Crosstalk between H3K4me3 and H3K14 acetylation has also been reported in *S. cerevisiae *(Maltby *et al*., 2012). ChIP analysis is now required to determine the methylation status of these lysine residues within the secondary metabolite BGCs of *C. higginsianum* in wild‐type and ∆*cclA* strains.

Until recently, almost nothing was known about the epigenetic regulation of secondary metabolism and pathogenicity in any species of the agriculturally important genus *Colletotrichum*. The only previous evidence comes from *C. fioriniae*, where treatment with the chemical epigenetic modifier trichostatin A, which inhibits histone deacetylases, was found to induce the production of cercosporin by mycelial cultures (de Jonge *et al*., 2018). By genetic manipulation of the COMPASS component *cclA*, we show here that H3K4 trimethylation plays an important role in the chromatin‐level regulation of secondary metabolism in *C. higginsianum*. This has enabled us to identify several novel compounds, highlighting the great potential of this approach for the discovery of new metabolites from *Colletotrichum *species. The overproduction of these molecules by mycelial cultures growing *in vitro* also allowed purification of these molecules in milligram quantities, which will facilitate future screening of their biological activities.

## Experimental Procedures

### Bacterial and fungal strains

*Escherichia coli* TOP10 was used as recipient for subcloning. Bacteria were maintained on LB medium supplemented with antibiotics when suitable. The strain IMI 349063 of *C. higginsianum* was used as the wild‐type strain and maintained on Mathur's medium as previously described (O'Connell *et al*., 2004).

### Generation of constructs and transformation

The gene replacement constructs were produced using the double‐joint PCR (DJ‐PCR) method (Szewczyk *et al*., 2006), subcloned into the pCR‐BluntII‐TOPO vector (450245, Invitrogen, Carlsbad, California) and propagated into *E. coli *TOP10. The transformation constructs were produced either using DJ‐PCR or Gateway technology. For targeted gene replacement of *cclA*, the 5' and 3' flanking regions were amplified with primers *5'flank‐cclA‐F* and *5'flank‐cclA‐R* (Supplementary Table S1). The *hph* gene fragment was amplified with primers *cclA‐TrpC‐F* and *cclA‐hph‐R *from plasmid pBIG4MRHrev (Tsuji *et al*., 2003). The three fragments were assembled with primers *nest‐cclA‐F* and *nest‐cclA‐R* and cloned as described above yielding pCRII‐KOcclA. Protoplasts of the wild‐type strain were transformed with the deletion cassette and transformants selected on PDA supplemented with 100 µg/mL hygromycin. In order to reintegrate the wild‐type gene at the mutant locus, the entire *cclA* locus was amplified by PCR from wild‐type genomic DNA with *nest‐cclA‐F* and *nest‐cclA‐R* primers. The selection marker (*Neo* gene) conferring geneticin (G418) resistance was provided by the plasmid pCGEN linearized with HpaI (Motteram *et al*., 2009). Protoplasts of the Δ*cclA* mutant were then co‐transformed with the wild‐type *cclA* locus and the selection marker using PEG‐mediated transformation as described by Dallery *et al*. (2017). Transformants were selected twice on PDA supplemented with 300 µg/mL G418. Confirmed reintegrants were purified by single‐spore subculture.

### Southern blot

Fungal genomic DNA was extracted as previously described (Huser *et al*., 2009). At least 8 µg of genomic DNA was digested to completion with SalI restriction enzyme. Digested DNA was blotted on Amersham N + nylon membrane (GE Healthcare, Chicago, Illinois, USA) using a vacuum blotter. Membranes were prehybridized with hybridization solution (5 × SSC, 20 mM maleic acid, 0.1% N‐lauroylsarcosin, 0.02% SDS [sodium dodecyl sulfate], 2% blocking reagent [Roche], 3 M urea) for 30 min at 50 °C. Probes were prepared following manufacturer's instructions with the PCR DIG Probe Synthesis Kit (11636090910, Roche, Mannheim, Germany) and used for hybridization at 50 °C overnight. Blots were washed twice with 2 × SSC/0.1% SDS at room temperature and twice with 0.5 × SSC/0.1% SDS at 65 °C. Detection was performed with autoradiography films using the DIG Luminescent Detection Kit according to the manufacturer's instructions (11363514910, Roche, Mannheim, Germany).

### Histone extraction and Western blot analysis

Histones were extracted as described by Honda and Selker (2008) with minor changes. Mycelium was grown in liquid Czapek‐Dox medium at 25 °C for 3 days, recovered by filtration and flash‐frozen in liquid nitrogen. Frozen tissue was ground with a mortar and pestle and 5 g was resuspended in 8 mL of ice‐cold lysis buffer (0.3 M sucrose, 40 mM NaHSO_3_, 25 mM Tris‐HCL [pH 7.4], 0.5 mM EDTA, 0.5% NP‐40, 1 mM PMSF, 5 µg/mL Leupeptin, 1 × Protease inhibitor cocktail [P8215, Sigma, Saint‐Louis, MO, USA]). Nuclei were pelleted by centrifugation (8000 × g, 4 °C, 15 min), washed with the same buffer and centrifuged again. Nuclei were resuspended in 4 mL of ice‐cold CW buffer (150 mM NaCl, 10 mM Tris‐HCL [pH 8], 1 mM 2‐mercaptoethanol, 1 mM PMSF, 5 µg/mL Leupeptin, 1 × Protease inhibitor cocktail). The subsequent steps were unchanged. Histones were quantified and then diluted in Laemmli's buffer. An equal amount of protein (25 µg) was separated by SDS‐PAGE (15% acrylamide gel) and subjected to western blotting using antibodies (from Active Motif, Carlsbad, CA, USA) raised against H3K4me1 (39297), H3K4me2 (39142), H3K4me3 (39159) and H3 C‐terminal fragment (39163) as a loading control. Anti‐mouse antibodies conjugated with Alkaline Phosphatase (AP, A90‐116AP, Bethyl, Montgomery, TX, USA) or anti‐rabbit antibodies conjugated with Horseradish Peroxidase (HRP, A0545, Sigma) were used as secondary antibodies. The Immobilon Substrate Kit was used for HRP detection (WBKLS0500, Merck, Darmstadt, Germany) and standard BCIP‐NBT substrate for AP detection. Blots were recorded with a ChemiDoc Imaging System (Bio‐Rad, Hercules, CA, USA).

### Developmental phenotyping

Four‐week‐old *A. thaliana* Col‐0 plants were spray‐inoculated with conidial suspension (5 × 10^5^ spores/mL) of the wild‐type strain or mutants as previously described (Huser *et al*., 2009). Methods for Trypan blue‐lactophenol staining of infected leaves and subsequent clearing of the stained tissues in chloral hydrate were adapted from Keogh *et al*. (1980). Quantification of the leaf area occupied by necrotic lesions was based on the analysis of at least six leaves from four plants in each of three independent inoculation experiments using ImageJ (Measure, Analyze Particles, version 1.51f) (Schneider *et al*., 2012). To quantify the frequency of appressorial penetration, Trypan blue‐stained *Arabidopsis* leaves were examined microscopically and appressorium penetration was scored as the presence of a visible hypha in the underlying epidermal cell. Mycelial growth rate *in vitro* was evaluated using CDA minimal medium or PDA for 10 days at 25 °C with at least seven replicates and three independent repetitions. Sporulation was quantified after 14 days growth on Mathur's medium at 25 °C (four replicates and three independent repetitions). To quantify spore germination and appressorium formation *in vitro*, polystyrene Petri dishes were sprayed with conidial suspension (5 × 10^5^ spores/mL) and examined by bright‐field microscopy after incubation for 24 h at 25 °C. To evaluate penetration of cellophane membranes, spores (1 × 10^6^ spores/mL) were inoculated onto pieces of autoclaved Visking dialysis tubing (Carl Roth GmbH, Karlsruhe, Germany) and viewed by phase‐contrast microscopy after incubation for 48 h at 25 °C.

### Metabolite profiling of wild‐type and mutant strains

For comparative metabolite profiling of the ∆*cclA* mutant and wild‐type strains, fungi were cultivated in PDB (Difco, Becton Dickinson and Company, Sparks, MD, USA) amended with 30 g/L of Amberlite® XAD 16 resin. For each strain, a mixture of spores and mycelium was used to inoculate one Erlenmeyer flask each containing 1 L of PDB medium and allowed to grow at 25 °C with shaking (150 rpm) for 5 days. At the end of the incubation period, fungal biomass and resin were recovered together and separated from the supernatant by filtration. The mixture of fungal biomass and resin was extracted with 100 mL of ethyl acetate, dried over Na_2_SO_4_ and evaporated under reduced pressure. Crude extracts were solubilized in the same amount of methanol and for each strain an equal volume of extract was analysed by HPLC. The experiment was repeated twice on different occasions.

### General chemistry procedures

HPLC was performed using an Alliance Waters 2695 controller coupled with an evaporative light‐scattering detector ELSD Waters 2424, a PhotoDiode Array Waters 2996 and a Waters QDa mass detector. The HPLC analytical column used was a 3.5 µm, C‐18 column (Sunfire 150 mm × 4.6 mm) operating at 0.7 mL/min. The gradient consisted of a linear gradient for 50 min from H_2_O to acetonitrile, both containing 0.1% formic acid. All other chemicals and solvents were purchased from SDS (France). Procedures related specifically to metabolite isolation, dereplication and structural elucidation are described elsewhere (Dallery *et al*., 2019).

## Supporting information


**Fig. S1** Phylogenetic tree of characterized Bre2 homologues and a *Colletotrichum higginsianum* homologue. Protein sequences were aligned using Muscle (v3.8.31), gaps were removed using Gblocks with relaxed parameters (v0.91b) and phylogeny was inferred using Maximum Likelihood implemented in PhyML (v3.0). Robustness was evaluated using the approximate likelihood ratio test (aLRT) and is indicated on each node. Branch length is proportional to the number of substitutions per site.

**Fig. S2** Genetic manipulation of the *cclA* gene. (A) Genomic location of the wild type (WT) *cclA *locus and the transformation cassette used for deleting the complete open reading frame (ORF). Relevant *Sal*I restriction sites are indicated (S). (B) Southern blot of the WT and Δ*cclA* mutants #18, #27 and #31. The probe used is depicted in Panel A. Expected sizes: WT   1.9 kb; at the locus insertion   3.2 kb.

**Fig. S3** Loss of CclA alters *C. higginsianum* spore morphology. (A) Ungerminated spores of the wild type (WT), *cclA* mutant and C9 complemented strain viewed with differential interference contrast microscopy using a 63× (NA 1.25) objective. Spores of the *cclA* mutant show greater morphological and size variability than the wild type and complemented strains, with more curved (white circle) or abnormally short (arrow) or long (arrowhead) spores present*.* Bars   20 µm. (B) Boxplot showing the lengths of spores of the WT, Δ*cclA* mutant and C9 complemented strains.

**Fig. S4** Appressorium morphogenesis and melanization are not impacted by deletion of *cclA*. Appressoria formed by conidia of the wild type strain (A) and *cclA* mutant (B,C) germinating on polystyrene for 24 h at 25 °C, viewed by bright field microscopy (40× NA 0.75). The arrow indicates a weakly melanized appressorium formed by an aberrantly small conidium. Bar    10 µm.

**Table S1** Primers used in this study.

**Table S2** Semi‐quantification of compounds in cultures of wild type and Δ*cclA *strains.
