# Supplementary material for: H3K4 trimethylation by CclA regulates pathogenicity and the production of three families of terpenoid secondary metabolites in Colletotrichum higginsianum
Source: Mol Plant Pathol. 2019 Mar 29;20(6):831–42. doi: 10.1111/mpp.12795 (PMC6637877; doi:10.1111/mpp.12795)
Supplement: Supplementary file 1 — Fig. S1 Phylogenetic tree of characterized Bre2 homologues and a Colletotrichum higginsianum homologue. Protein sequences were aligned using Muscle (v3.8.31), gaps were removed using Gblocks with relaxed parameters (v0.91b) and phylogeny was inferred using Maximum Likelihood implemented in PhyML (v3.0). Robustness was evaluated using the approximate likelihood ratio test (aLRT) and is indicated on each node. Branch length is proportional to the number of substitutions per site. [file MPP-20-831-s001.docx]

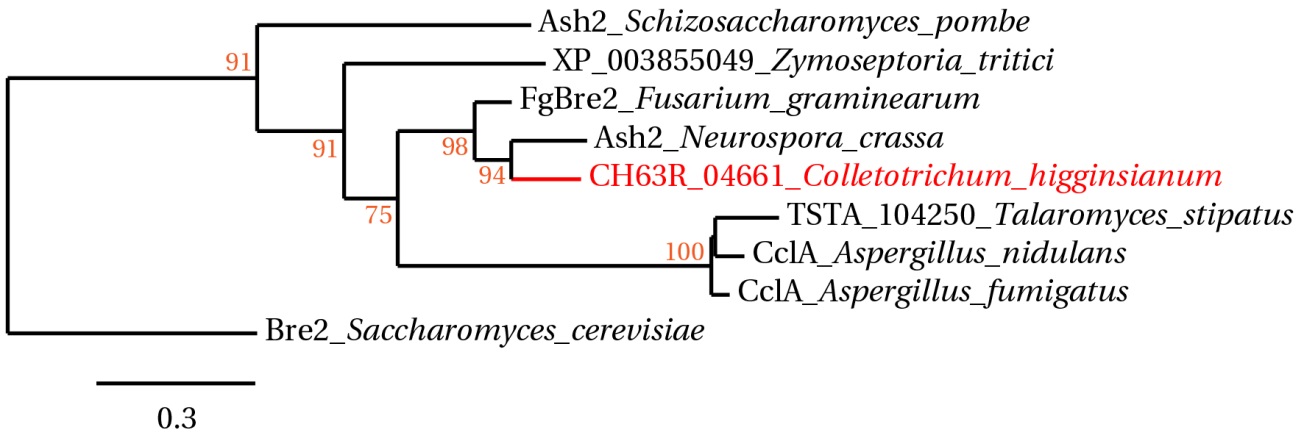


**Supplementary Figure S1: Phylogenetic tree of characterized Bre2 homologues and a *Colletotrichum higginsianum* homolog.** Protein sequences were aligned using Muscle (v3.8.31), gaps were removed using Gblocks with relaxed parameters (v0.91b) and phylogeny was inferred using Maximum Likelihood implemented in PhyML (v3.0). Robustness was evaluated using the approximate likelihood-ratio test (aLRT) and is indicated on each node. Branch length is proportional to the number of substitutions per site.
