# Supplementary material for: H3K4 trimethylation by CclA regulates pathogenicity and the production of three families of terpenoid secondary metabolites in Colletotrichum higginsianum
Source: Mol Plant Pathol. 2019 Mar 29;20(6):831–42. doi: 10.1111/mpp.12795 (PMC6637877; doi:10.1111/mpp.12795)
Supplement: Supplementary file 2 — Fig. S2 Genetic manipulation of the cclA gene. (A) Genomic location of the wild type (WT) cclA locus and the transformation cassette used for deleting the complete open reading frame (ORF). Relevant SalI restriction sites are indicated (S). (B) Southern blot of the WT and ΔcclA mutants #18, #27 and #31. The probe used is depicted in Panel A. Expected sizes: WT 1.9 kb; at the locus insertion 3.2 kb. [file MPP-20-831-s002.docx]

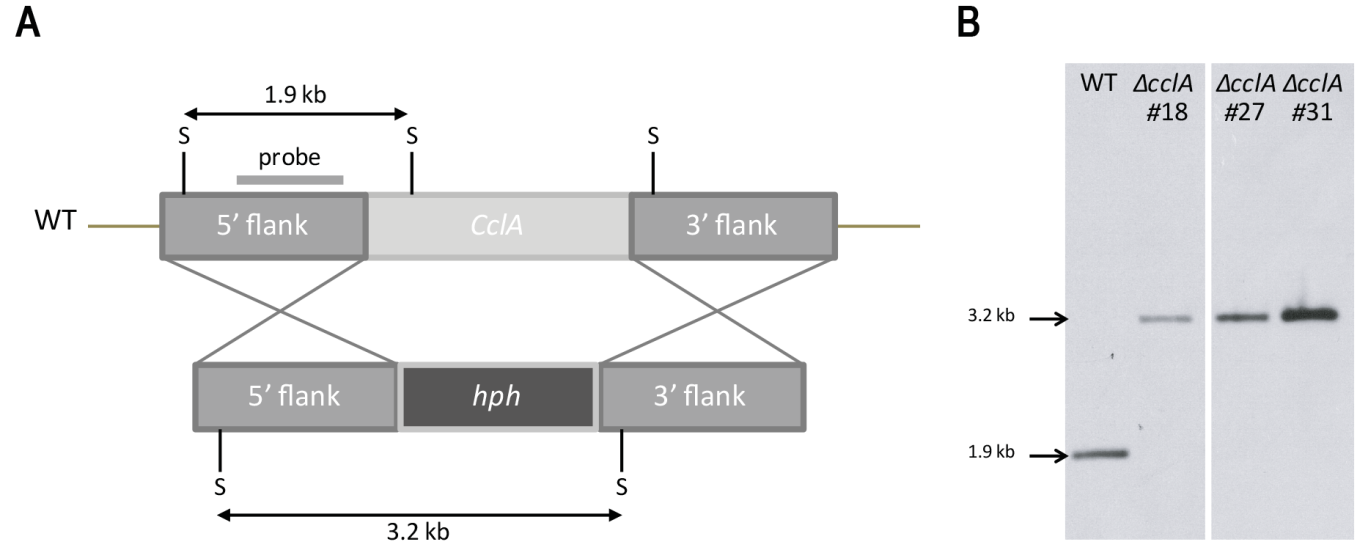


**Supplementary Figure S2: Genetic manipulation of the *cclA* gene. (A)** Genomic location of the wild-type (WT) c*clA* locus and the transformation cassette used for deleting the complete ORF. Relevant *Sal*I restriction sites are indicated (S). **(B)** Southern blot of the WT and *ΔcclA* mutants #18, #27 and #31. The probe used is depicted in panel A. Expected sizes: WT = 1.9 kb; at-the-locus insertion = 3.2 kb.
