# Supplementary material for: H3K4 trimethylation by CclA regulates pathogenicity and the production of three families of terpenoid secondary metabolites in Colletotrichum higginsianum
Source: Mol Plant Pathol. 2019 Mar 29;20(6):831–42. doi: 10.1111/mpp.12795 (PMC6637877; doi:10.1111/mpp.12795)
Supplement: Supplementary file 3 — Fig. S3 Loss of CclA alters C. higginsianum spore morphology. (A) Ungerminated spores of the wild type (WT), cclA mutant and C9 complemented strain viewed with differential interference contrast microscopy using a 63× (NA 1.25) objective. Spores of the cclA mutant show greater morphological and size variability than the wild type and complemented strains, with more curved (white circle) or abnormally short (arrow) or long (arrowhead) spores present. Bars 20 µm. (B) Boxplot showing the lengths of spores of the WT, ΔcclA mutant and C9 complemented strains. [file MPP-20-831-s003.docx]

**
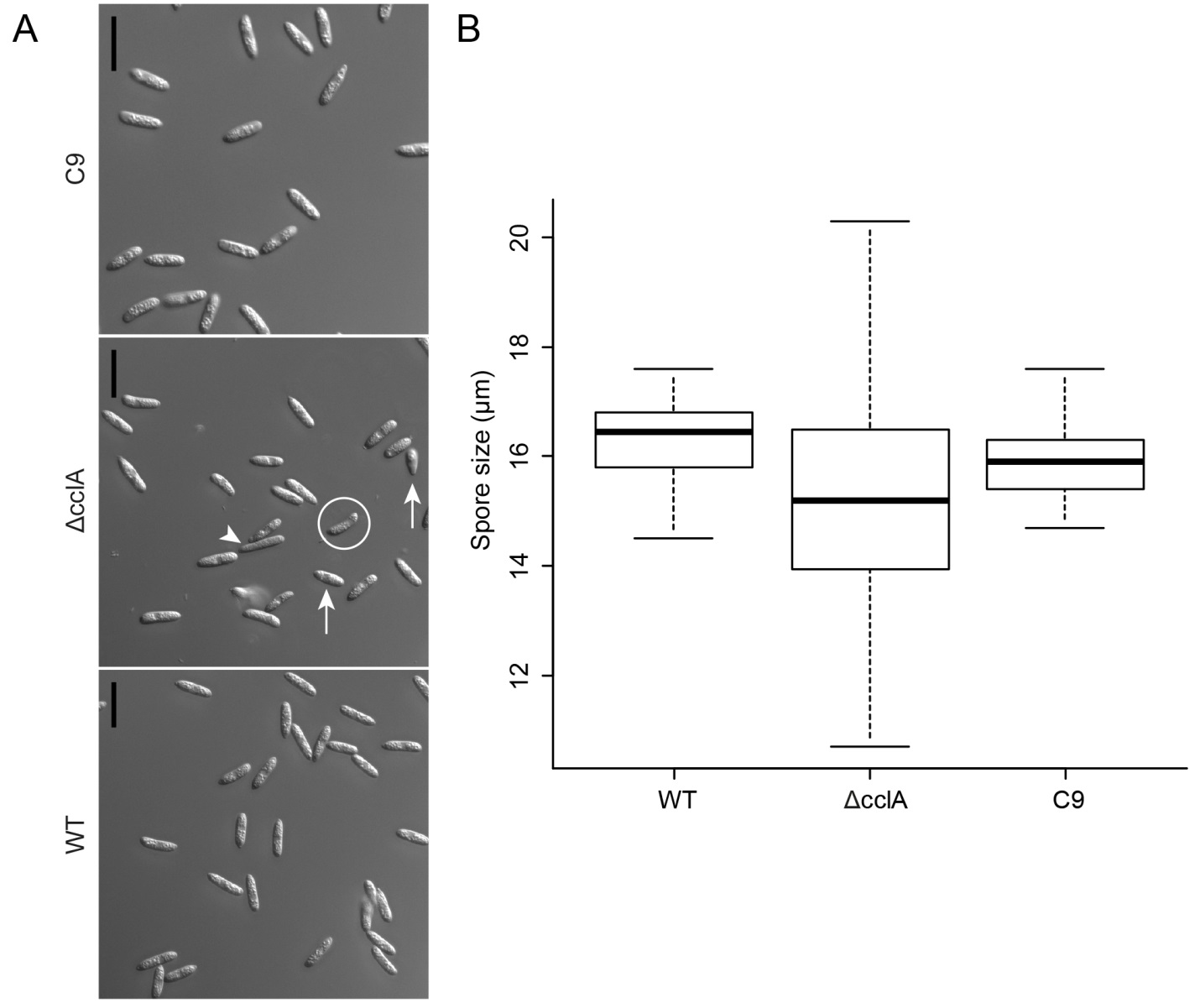
**

**Supplementary Figure S3: Loss of CclA alters *C. higginsianum* spore morphology. (A)** Ungerminated spores of the wild-type (WT), Δ*cclA* mutant and C9 complemented strain viewed with differential interference contrast microscopy using a 63x (NA 1.25) objective. Spores of the Δ*cclA* mutant show greater morphological and size variability than the wild-type and complemented strain, with more curved (white circle) or abnormally short (arrow) or long (arrowhead) spores present*.* Bars = 20 µm. **(B)** Boxplot showing the lengths of spores of the WT, *ΔcclA* mutant and C9 complemented strains.
