# Supplementary material for: H3K4 trimethylation by CclA regulates pathogenicity and the production of three families of terpenoid secondary metabolites in Colletotrichum higginsianum
Source: Mol Plant Pathol. 2019 Mar 29;20(6):831–42. doi: 10.1111/mpp.12795 (PMC6637877; doi:10.1111/mpp.12795)
Supplement: Supplementary file 4 — Fig. S4 Appressorium morphogenesis and melanization are not impacted by deletion of cclA. Appressoria formed by conidia of the wild type strain (A) and cclA mutant (B,C) germinating on polystyrene for 24 h at 25 °C, viewed by bright field microscopy (40× NA 0.75). The arrow indicates a weakly melanized appressorium formed by an aberrantly small conidium. Bar 10 µm. [file MPP-20-831-s004.docx]

**
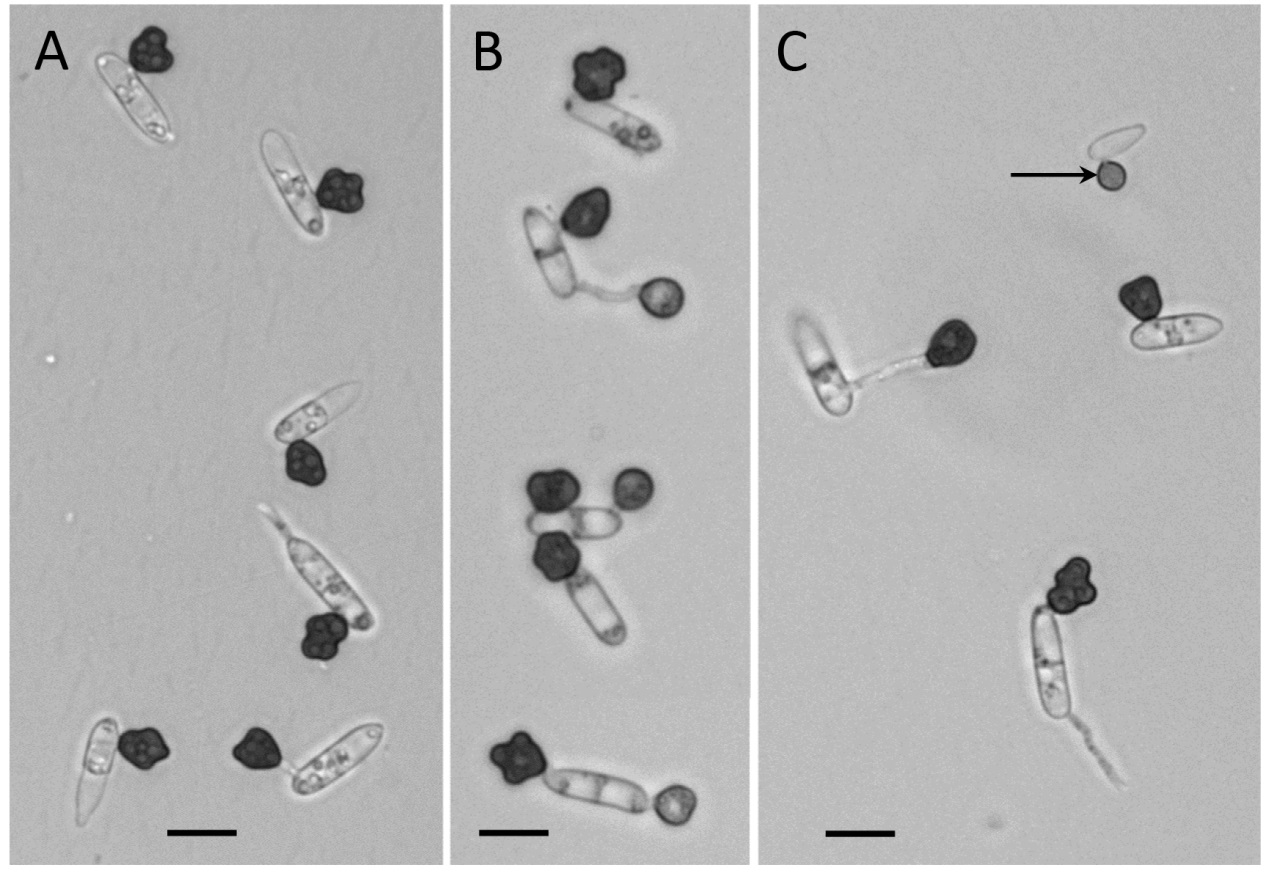
**

**Supplementary Figure S4: Appressorium morphogenesis and melanisation are not impacted by deletion of *cclA*.** Appressoria formed by spores of the wild-type **(A)** and Δ*cclA* mutant **(B,C)** germinating on polystyrene for 24 h at 25 °C, viewed by bright-field microscopy (40x/NA 0.75). Bars = 20 µm.
