# Supplementary material for: H3K4 trimethylation by CclA regulates pathogenicity and the production of three families of terpenoid secondary metabolites in Colletotrichum higginsianum
Source: Mol Plant Pathol. 2019 Mar 29;20(6):831–42. doi: 10.1111/mpp.12795 (PMC6637877; doi:10.1111/mpp.12795)
Supplement: Supplementary file 5 — Table S1 Primers used in this study. [file MPP-20-831-s005.docx]

**Supplementary Table S1: Primers used in this study.**

| **Designation** | **Sequence 5' to 3'** | **Restriction site** | **Notes** |
| --- | --- | --- | --- |
| 5'flank cclA-F1 | ACAAATCCACGCGACAATCA |  | 5' flanking region of c*clA* |
| 5'flank cclA-R1 | caatatcagttaacGAGTCGGACGCCATTGAATAG |  |  |
| 3'flank cclA-F2 | gcaaaggaatag**ctcgag**TCAGAGCGATACACGGAGCAGATT | ***Xho*I** | 3' flanking region of *cclA* |
| 3'flank cclA-R2 | GGGAGAGGAGGCAGAGACCAGTA |  |  |
| cclA-TrpC-F | ggcgtccgactcGTTAACTGATATTGAAGGAGCAT |  | Marker of selection |
| cclA-hph-R | ctctga**ctcgag**CTATTCCTTTGCCCTCGGA | ***Xho*I** |  |
| Nest-cclA-F | **ggcgcgcc**GAGGGACAGACGGGAGGACTTTTG | ***Asc*I** | Nested primers |
| Nest-cclA-R | **ggcgcgcc**GCTTAGGTGCGGGCTGTTTGA | ***Asc*I** |  |
| Int-hph-F | CGTTGCAAGACCTGCCTGAA |  | Internals to *hph* ORF |
| Int-hph-R | GGATGCCTCCGCTCGAAGTA |  |  |
| Int-neo-F | TATTCGGCTATGACTGGGCAC |  | Internals to *neo* ORF |
| Int-neo-R | TCTTCAGCAATATCACGGGTAG |  |  |
| Int-cclA-F | GGACTCTCCCCAGCTCTTAC |  | Internals to *cclA* ORF |
| Int-cclA-R | GTCGATCATGGAGACCTCGT |  |  |
