# Supplementary material for: H3K4 trimethylation by CclA regulates pathogenicity and the production of three families of terpenoid secondary metabolites in Colletotrichum higginsianum
Source: Mol Plant Pathol. 2019 Mar 29;20(6):831–42. doi: 10.1111/mpp.12795 (PMC6637877; doi:10.1111/mpp.12795)
Supplement: Supplementary file 6 — Table S2 Semi‐quantification of compounds in cultures of wild type and ΔcclA strains. [file MPP-20-831-s006.docx]

**Supplementary Table S2: Semi-quantification of compounds in cultures of wild-type and Δ*cclA* strains**

|  | **Peak area*** | | **Fold-increase** |
| --- | --- | --- | --- |
| **Compound** | **Wild-type** | **Δ*cclA*** | **in mutant** |
| higginsianin C | 0 | 24 | Only detected in Δ*cclA* |
| *13-epi*-higginsianin C | 0 | 11 | Only detected in Δ*cclA* |
| colletorin D acid | 0 | 11 | Only detected in Δ*cclA* |
| colletorin D | 0 | 11 | Only detected in Δ*cclA* |
| sclerosporide | 0 | 10 | Only detected in Δ*cclA* |
| colletorin A | 0.33 | 23 | 70 |
| higginsianin A | 1.5 | 62 | 41 |
| colletochlorin B | 0.1 | 3 | 30 |
| colletochlorin D | 1 | 27 | 27 |
| colletochlorin A | 3 | 41 | 14 |
| higginsianin B | 23 | 167 | 7 |
| eremophilane | 2.8 | 2.9 | 1 |

*Measured from original chromatograms
